# Proteomics of Homeobox7 Enhanced Salt Tolerance in *Mesembryanthemum crystallinum*

**DOI:** 10.3390/ijms22126390

**Published:** 2021-06-15

**Authors:** Xuemei Zhang, Bowen Tan, Dan Zhu, Daniel Dufresne, Tingbo Jiang, Sixue Chen

**Affiliations:** 1 State Key Laboratory of Tree Genetics and Breeding, Northeast Forestry University, Harbin 150040, China; zhangxuemei199111@gmail.com; 2 Department of Biology, Genetics Institute, University of Florida, Gainesville, FL 32610, USA; tanbowen@ufl.edu (B.T.); zhudan2014dora@163.com (D.Z.); 3 College of Life Sciences, Qingdao Agricultural University, Qingdao 266109, China; 4 Department of Chemistry, Florida Atlantic University, Boca Raton, FL 33431, USA; dufresne71@yahoo.com; 5 Plant Molecular and Cellular Biology Program, University of Florida, Gainesville, FL 32610, USA; 6 Proteomics and Mass Spectrometry, Interdisciplinary Center for Biotechnology Research, University of Florida, Gainesville, FL 32610, USA

**Keywords:** *Mesembryanthemum crystallinum*, *McHB7*, transcription factor, salt stress, proteomics

## Abstract

*Mesembryanthemum crystallinum* (common ice plant) is a halophyte species that has adapted to extreme conditions. In this study, we cloned a *McHB7* transcription factor gene from the ice plant. The expression of *McHB7* was significantly induced by 500 mM NaCl and it reached the peak under salt treatment for 7 days. The *McHB7* protein was targeted to the nucleus. *McHB7*-overexpressing in ice plant leaves through *Agrobacterium*-mediated transformation led to 25 times more *McHB7* transcripts than the non-transformed wild type (WT). After 500 mM NaCl treatment for 7 days, the activities of superoxide dismutase (SOD) and peroxidase (POD) and water content of the transgenic plants were higher than the WT, while malondialdehyde (MDA) was decreased in the transgenic plants. A total of 1082 and 1072 proteins were profiled by proteomics under control and salt treatment, respectively, with 22 and 11 proteins uniquely identified under control and salt stress, respectively. Among the 11 proteins, 7 were increased and 4 were decreased after salt treatment. Most of the proteins whose expression increased in the *McHB7* overexpression (OE) ice plants under high salinity were involved in transport regulation, catalytic activities, biosynthesis of secondary metabolites, and response to stimulus. The results demonstrate that the *McHB7* transcription factor plays a positive role in improving plant salt tolerance.

## 1. Introduction

*Mesembryanthemum crystallinum* (common ice plant) is a well-known halophyte plant native to Africa, southern Europe, and widely naturalized elsewhere [1]. It has been used as a health-promoting vegetable against diabetes [2]. Also, ice plant has been increasingly used as a model for studying abiotic stress responses [1] because it can shift from C3 photosynthesis to crassulacean acid metabolism (CAM) [3]. Halophytes, such as ice plants, tolerate high salinity by accumulating osmolytes in specific locations and balancing ion homeostasis, thereby maintaining osmolarity [4]. In ice plants, the special epidermal bladder cells (EBCs) on the leaf surface help to sequester salt [5]. The mesophyll cells and diurnal regulation of stomatal guard cells also improve water use efficiency of the ice plants [6].

Recent studies have revealed changes in ice plants at transcriptomic [7,8], intracellular ion [9], metabolite [10], and protein levels [11] in response to salt stress, with many candidate genes, proteins and metabolites of relevance having been identified. For example, *McSnRK1* encodes a SNF1-related protein kinase, which functions as transcriptional modulator to control metabolic adaption, regulate Na^+^ flux and maintain Na/K homeostasis under salt stress [12]. Transgenic tobacco co-expressing an ice plant inositol methyl transferase gene, *McIMT1,* and a wild halophytic rice gene, *PcINO1,* accumulated high levels of inositol and methylated inositol. These metabolites may help to improve plant tolerance to high degrees of salt stress [13]. A sodium transporter, *McHKT2*, was reported to participate in plasma membrane Na^+^ transport and induce significant salt tolerance in transgenic *Arabidopsis* *thaliana* (*A. thaliana*) seedlings [14]. In addition, Kong et al. found that the transition from C3 to CAM photosynthesis takes place between day 5 and day 7 after treating ice plants with 500 mM NaCl. A total of 495 transcripts were significantly changed in guard cells based on RNA-Seq data analysis [15]. Among them, 18 transcription factors (TFs) were identified, including NAC (comp21521_c0_seq1 and Mcr002150.001), GRAS (Contig16446), WRKY (Contig20720), and homeobox family TFs (Contig9771, *McHB7*).

Homeobox genes have been found in all eukaryotic organisms [16]. They have a highly conserved homeodomain (HD), which contains a specific 60 amino acids (aa) DNA-binding motif. The HD forms a helix-turn-helix structure that regulates the expression of target genes [17,18]. The HD is always followed by a leucine zipper motif (35–42 aa), termed as homeobox associated leucine zipper (HALZ), which functions as a dimerization motif associated with the HD [19]. Homeobox genes are master regulators of plant development [20], meristem regulation [20], hormone mediation [21], and response to various environmental stimuli—including high salinity, drought, and extreme temperature [22]. For example, STIMPY/AtWOX9 was found to be important to the growth of vegetative shoot apical meristems and the maintenance of cell division in the shoot and root apex [23]. Overexpressing a poplar WUSCHEL-related homeobox gene, *PagWOX11*/*12a*, increased plant root biomass and enhanced drought tolerance [24,25]. *WOX6*/*HOS9* is important for low temperature response in Arabidopsis by mediating the expression of genes independent of element-binding factor pathway [26]. In upland cotton, *GhWOX10_Dt*, *GhWOX13b_At*/*Dt*, and *GhWOX13a_At*/*Dt* were significantly induced by salt stress, and they may play vital roles in improving salt stress response of plants [27].

In Kong et al. [15], it was reported that *McHB7* in ice plant was induced by 500 mM NaCl treatment for 7 days during the transition from C3 to CAM. Here we report additional results on *McHB7* functions. Although it is common to analyze gene functions by genetic transformation, transformation of ice plants to study the functions of salinity-related genes is rare. The objective of this study is to analyze the ice plant TF *McHB7* and its function in salt stress tolerance. We cloned the *McHB7* from ice plant leaves and examined the molecular changes in transiently transformed plants compared to WT under control and high salinity conditions.

## 2. Results

### 2.1. Bioinformatic Analysis of McHB7

**Figure 1:**
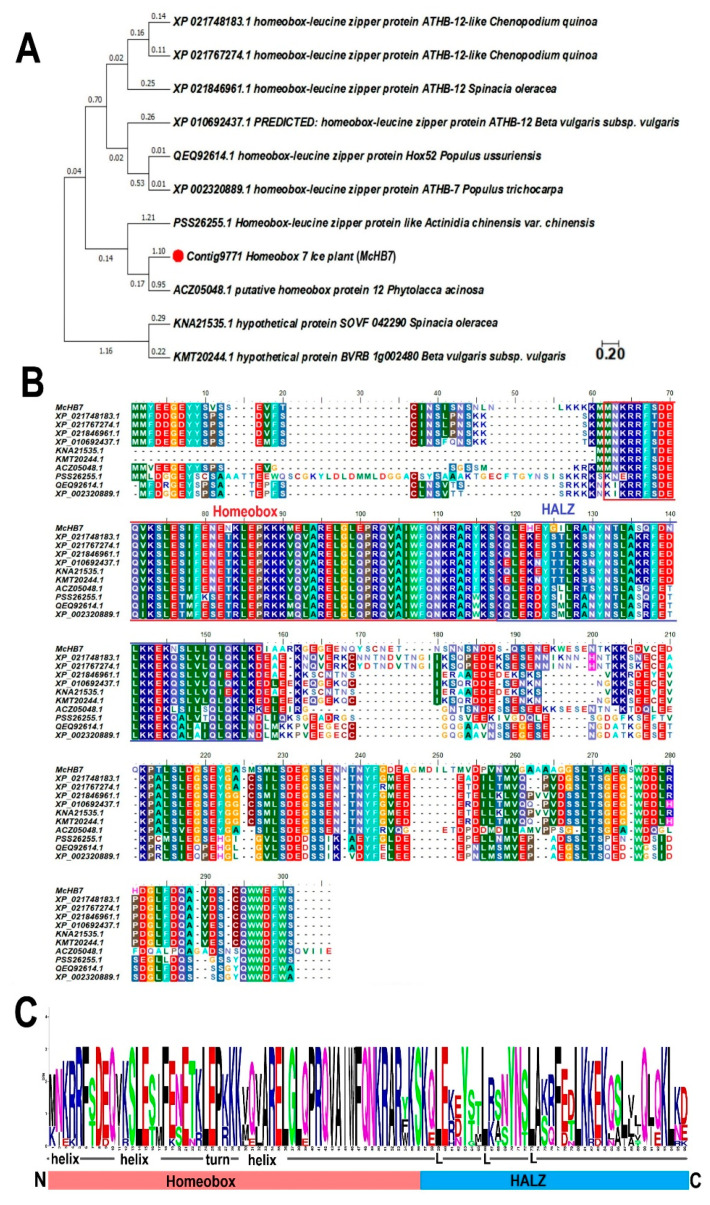
Bioinformatic analysis of *McHB7* and homologous TF proteins. (**A**) Phylogenetic tree of XP_021748183.1, XP_021767274.1, XP_021846961.1, XP_010692437.1, KNA21535.1, KMT20244.1, ACZ05048.1, PSS26255.1, QEQ92614.1, and XP_002320889.1. The red dot indicates the *McHB7* TF. (**B**) Amino acid sequence alignment of XP_021748183.1, XP_021767274.1, XP_021846961.1, XP_010692437.1, KNA21535.1, KMT20244.1, ACZ05048.1, PSS26255.1, QEQ92614.1, and XP_002320889.1. (**C**) Two domains of homeobox TFs: conserved homeobox domain (HD) and variable HALZ domain.

As shown in Supplementary Figure S1, the cloned *McHB7* gene is 804 bp and it encodes a TF of 267 amino acids. According to NCBI blast, 10 homologous proteins from other species shared high homology, i.e., XP_021748183.1 (64.1%, *Chenopodium quinoa*), XP_021767274.1 (64.0%, *Chenopodium quinoa*), XP_021846961.1 (61.1%, *Spinacia oleracea*), XP_010692437.1 (61.6%, *Beta vulgaris subsp. vulgaris*), KNA21535.1 (61.1%, *Spinacia oleracea*), KMT20244.1 (61.7%, *Beta vulgaris subsp. vulgaris*), ACZ05048.1 (54.8%, *Phytolaccaacinosa*), PSS26255.1 (48.4%, *Actinidia chinensis var. chinensis*), QEQ92614.1 (48.9%, *Populusussuriensis*), and XP_002320889.1 (48.9%, *Populustrichocarpa*). Phylogenetic analysis (Figure 1A) and sequence alignment (Figure 1B) showed that these proteins contain two highly conserved domains: homeobox and homeobox associated HALZ. The basic region homeobox at the N terminal (Figure 1C) consists of three α-helices that form a helix-turn-helix DNA-binding motif, followed by a HALZ that is markedly different from those homeobox TFs in animal systems [19].

### 2.2. McHB7 TF Protein Localized to the Nucleus

**Figure 2:**
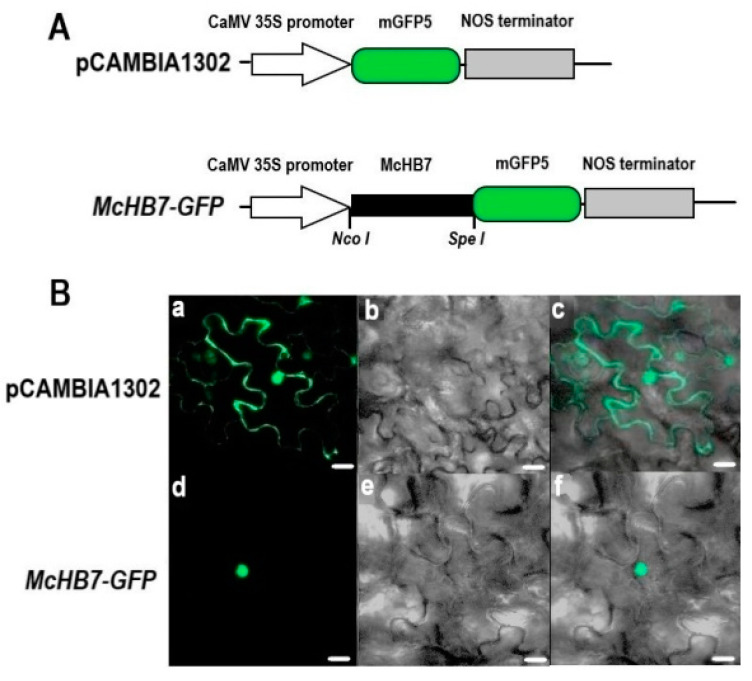
Subcellular localization of *McHB7* protein. (**A**) Schematic maps of the positive control pCAMBIA1302-*GFP* and the *McHB7*-*GFP* construct in the pCAMBIA1302vector. (**B**) The *McHB7*-*GFP* fusion construct and the positive control pCAMBIA1302were introduced into tobacco leaves by infiltration. *GFP* fluorescence was observed by confocal laser scanning microscopy. (**a**,**d**) fluorescence images observed in a dark field (green); (**b**,**e**) light images observed in bright field; (**c**,**f**) merged images of dark field and bright field. Scale bar = 30 μm.

CELLO2GO prediction showed the localization of *McHB7* protein to the nucleus (Supplementary Figure S2). To validate this predicted result, *A. tumefaciens* GV3101 containing an *McHB7*-*GFP* construct (Figure 2A) and the positive control pCAMBIA1302-*GFP* were transiently expressed in tobacco leaves. As shown in Figure 2B, *GFP* signal of the positive control was observed in the cytosol and nucleus of whole epidermal cell, while the *GFP* signal of *McHB7*-*GFP* only existed in the nucleus. This result clearly showed the nuclear localization of the *McHB7* TF.

### 2.3. Relative Expression of McHB7 in Ice Plant Leaves after Salt Stress Treatment

**Figure 3:**
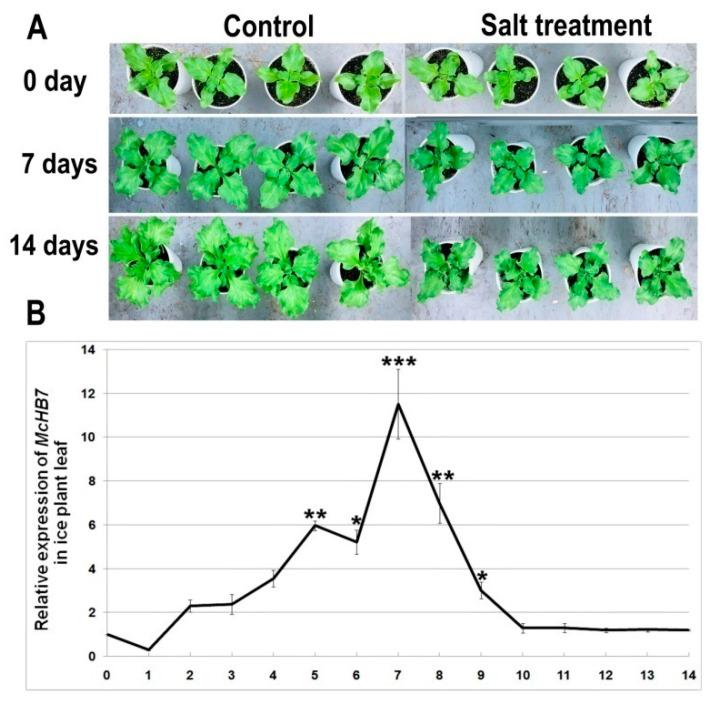
Ice plant growth phenotype and *McHB7* expression after high salinity treatment. (**A**) Phenotype of ice plant seedlings under 500 mM NaCl treatment for 0, 7, and 14 days. (**B**) Relative expression of *McHB7* after 500 mM NaCl treatment (compared with the control condition). Student’s *t*-test: *t*: * *p* < 0.05; ** *p* < 0.01, *** *p* < 0.001, error bars indicate mean ± standard deviation (SD) (*n* = 4).

To investigate the expression pattern of *McHB7* under high salinity, we treated the four-week-old ice plant seedlings with 500 mM NaCl and collected leaf samples each day for 14 days. When ice plants were salt-stressed for seven or 14 days, the growth was severely inhibited and the leaves were smaller when compared to the control seedlings (Figure 3A), and fresh weight of salt-treated leaves for seven days and 14 days was 49.9% and 24.9% that of control leaves, respectively (Supplementary Figure S3). According to the RT-qPCR results in Figure 3B, the relative expression of *McHB7* in ice plant leaves was significantly induced by salt stress, reached the peak at day 7, up to almost 12 times higher than untreated leaves.

### 2.4. Overexpressing McHB7 in Transgenic Ice Plants

**Figure 4:**
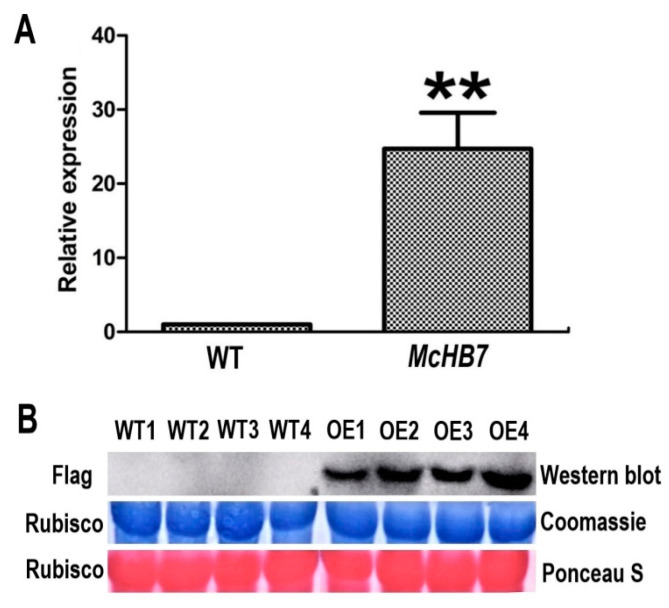
Overexpression of *McHB7* in ice plants at day 7 after transformation. (**A**) The relative expression of *McHB7* in the overexpression (OE) plants compared to WT; Student’s *t*-test, ** *p* < 0.01. Error bars indicate mean ±SD (*n* = 4). (**B**) Images of Western blot showing Flag signal of *McHB7* protein level in the OE plants. The protein loading was assessed by Coomassie staining and Ponceau S staining of Rubisco, showing equal loading. WT1–WT4 are four replicates of WT, OE1–OE4 are four replicates of *McHB7*-over-expressing seedlings.

To verify the OE of *McHB7* in the ice plant leaves, we performed RT-qPCR with *McHB7*-overexpressing (OE) plants and WT leaves. As shown in Figure 4A, 7 days after infiltration, the relative expression of *McHB7* in OE ice plant was significantly higher (~25 times) than WT. According to the Western blot analysis, the Flag signal only existed in the *McHB7*-infiltrated leaves (Figure 4B). These data clearly showed successful OE of *McHB7* at both the transcriptional and translational levels. However, there was no significant difference in SOD and POD activity or water content between OE plants and WT. MDA content of OE plants was a little lower than WT, but not statistically significant (Supplementary Figure S4).

### 2.5. Changes in Biochemical Parameters in Ice Plants after Salt Stress Treatment

**Figure 5:**
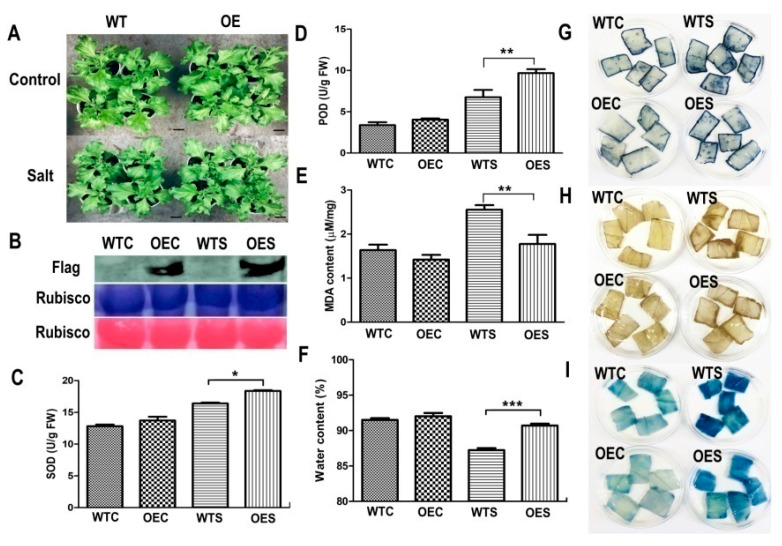
*McHB7*-overexpressing (OE) plants under control and salt stress conditions. (**A**) Phenotype of OE plants and WT under control and salt stress conditions, bar = 2 cm. (**B**) Validation of OE plants after salt stress treatment. The OE plants were at day 14 after transformation; Student’s *t*-test, * *p*< 0.05, ** *p* < 0.01, *** *p* < 0.001. Error bars indicate mean ± SD (*n* = 4). (**C**) SOD activity of OE plants and WT under control and salt stress conditions. (**D**) POD activity. (**E**) MDA content. (**F**) Water content. (**G**) NBT staining. (**H**) DAB staining. (**I**) Evans blue staining. WTC, WT under control conditions; OEC, transgenic plants under control conditions; WTS, WT after salt stress treatment; OES, transgenic plants after salt stress treatment.

One week after infiltration, the OE ice plant seedlings and WT were treated with 500 mM NaCl solution for another week (14 days after infiltration). As shown in Figure 5A, there were no significantly differences between the *McHB7*-overexpressingplants and WT under control conditions. After high salinity treatment for one week, the leaves of OE plants grew better than WT, with a fresh weight 1.2 times higher (Supplementary Figure S5). To further test whether the transgene is still functional, we carried out the Western blot with transgenic leaves under control and salt-treated conditions. The results showed that the Flag signal still existed in OE leaves 14 days after infiltration (Figure 5B). The control and OE leaves were collected for physiological analyses and histochemical staining. As described in Figure 5C–F, SOD activity, POD activity, and water content in OE were 1.2 ± 0.2, 1.5 ± 0.8, and 1.1 ± 0.5 times higher than in WT, respectively. Nitrotetrazoliumblue chloride (NBT) and3, 3′-diaminobenzidine (DAB) were used to analyze the levels of superoxide anions (O_2_^−^) and hydrogen peroxide (H_2_O_2_), respectively [28]. Evans blue was used to check cell death due to plasma membrane damage [29]. The staining intensities of OE and WT leaves were similar under control conditions (Figure 5G–I). After 500 mM NaCl treatment for seven days, the staining of WT was darker than the OE plants, and the relative intensity of NBT and DAB in WT was significantly higher than that of OE (Supplementary Figure S6). The result indicates overexpressing *McHB7* led to lower accumulation of reactive oxygen species (ROS) and stronger capability for ROS removal.

### 2.6. Proteomic Changes Attributed to McHB7 Overexpression and Salinity Treatment

**Figure 6:**
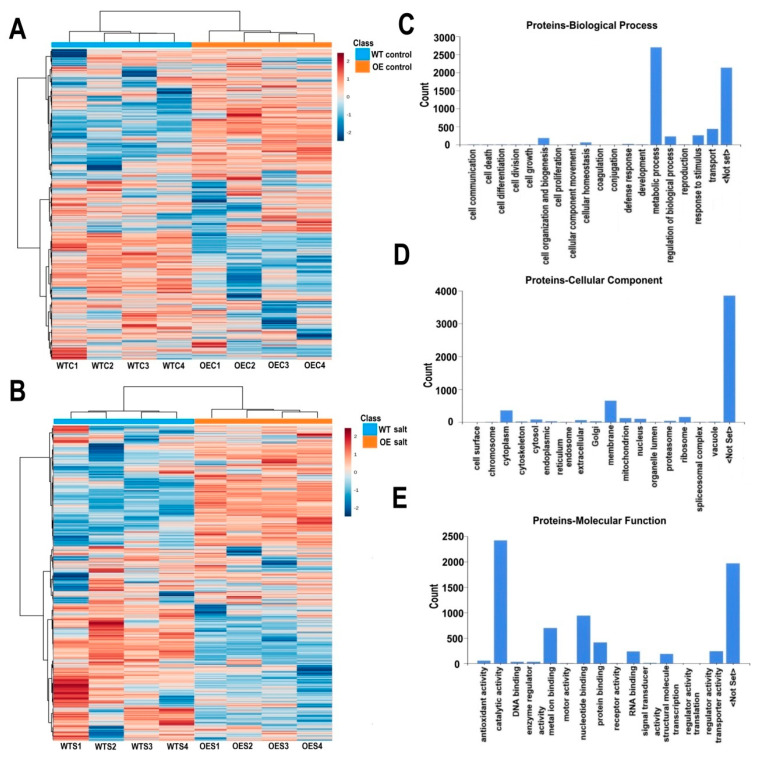
Heatmap of identified proteins under control and high salinity conditions and GO functional categorization of the proteins. (**A**) Identified proteins under control conditions. WTC1–WTC4, four biological replicates of WT under control conditions; OEC1–OEC4, four replicates of OE plants under control conditions. (**B**) Identified proteins under salt stress conditions. WTS1–WTS4, four biological replicates of WT after salt stress treatment; OES1–OES4, four biological replicates of transgenic plants after salt stress treatment. (**C**) Biological process of identified proteins. (**D**) Cellular component of identified proteins. (**E**) Molecular function of identified proteins.

Total proteins of OE and WT plant leaves were isolated and label-free quantitative proteomics was carried out using LC-MS/MS. Under control and salt stress conditions, 1082 and 1072 proteins were identified, respectively (Figure 6A,B). GO functional classifications including biological process, cellular component and molecular function were generated by Proteome Discoverer. Of the biological processes, most proteins were involved in metabolic process, transport, stimulus response or regulation (Figure 6C). Most of them were categorized to the cytoplasm, membrane, ribosome, mitochondrion, and nucleus (Figure 6D). Many proteins played a role in catalytic activity, nucleotide, and metal ion binding. Some of them were involved in protein and RNA binding (Figure 6E). Among these identified proteins, 34 proteins had phosphorylation modifications under control and salt stress conditions (Supplementary Table S2). Most of them were chlorophyll a-b binding proteins, which belong to the light-harvesting complex and could be photo-regulated through reversible phosphorylation. These were reported to mediate the distribution of excitation energy in the photosystems I and II [30].

### 2.7. Differentially Expressed Proteins under Control and Salt Stress Treatment

**Figure 7:**
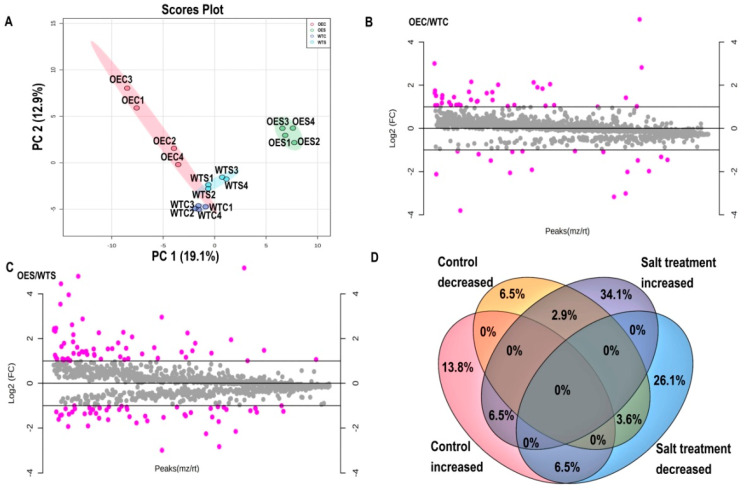
Differentially expressed proteins (DEPs) under control and salt stress treatment. WTC1-WTC4, four biological replicates of WT under control conditions; OEC1-OEC4, four replicates of OE plants under control conditions; WTS1-WTS4, four biological replicates of WT after salt stress treatment; OES1-OES4, four biological replicates of transgenic plants after salt stress treatment. (**A**) PCA result of all the control and salt stress samples of WT and *McHB7* OE. (**B**) DEPs under control conditions. (**C**) DEPs under salt stress treatment. (**D**) Venn diagram of DEPs under control and salt stress treatment.

To address the molecular mechanisms underlying *McHB7* function, we conducted proteomics on WT and OE plants under control and salt stress conditions. Principal component analysis (PCA) is an unsupervised multivariate statistics-based detection method to reveal the differences and relationships between samples [31]. In this study, four replicates of each sample were grouped together, but different samples were classified into distinct clusters (Figure 7A). The results showed that samples from OE and WT leaves under control and salt treatment, respectively, occupied relatively independent spaces in the distribution map, especially OES was separated and far away from WTS.

Based on the identified proteins from OE and WT leaves, differentially expressed proteins (DEPs) were analyzed. There were 55 proteins—37 increased and 18 decreased under control conditions (Figure 7B; Supplementary Figure S7A). Among these, seven proteins were experienced fold change (OEC/WTC) greater than four magnitudes. Notably, K9NCW5, a ribulose bisphosphate carboxylase/oxygenase (Rubisco) homologue was about 33 times higher in OE. Other increased proteins like A0A022RVT2, A0A1S3 × 8Z6, B9GEL5, A0A200R0A6, A0A072UU34, and M1C498 were involved in nucleotide binding that may interact with the nuclear localized *McHB7*. After high salinity treatment, there were 110 proteins including 60 increased and 50 decreased (Figure 7C; Supplementary Figure S7B). Among these increased proteins, six including D7KSJ9, A0A1J3DHL5, F4Y5A9, A0A1D6LMX1, A9TQU1, and A0A1Q3C6B9 were responsive to stimulus and were all involved in metabolic process. Besides, some proteins like A2TJU5 were proteins sensitive to high salinity and played a role in transport activities. W8E1S1 and A0A0F7GYT3 are photosystem II CP43 reaction center protein and photosystem II reaction center PsbP family protein, respectively. Their induction by salt may help maintain photosynthesis activity under the stress condition. A0A022RMX1, A0A1J3DHL5 and A9PAY7 were involved in regulation of biological activities, A0A022RMX1 and A0A1J3DHL5 took a part in the cell organization and biogenesis, A9PAY7 worked in cellular homeostasis. A0A0D2Q010 and M0U9P1 were involved in biosynthesis of secondary metabolites, A0A0D2Q010 played a role in citrate acid cycle, and M0U9P1was involved in glycine, serine, and threonine metabolism. A Venn Diagram shows that nine proteins were increased in *McHB7*-overexpressing leaves and after high salinity treatment (Figure 7D). Of these, the relative expression of K9NCW5, A0A178UQY1, A0A2P5EU89, A0A022RVT2, A0A078G853, and D7KSJ9 was 33.09, 8.04, 3.24, 2.89, 2.31, and 2.06 times higher in OE vs. WT, respectively, under control conditions. However, they were elevated to 35.65, 21.84, 6.1298, 3.62, 3.46, and 11.62 times, respectively, under high salinity. Interestingly, A0A075M528, A0A022RT96, A0A2J6MJ80, and A0A2R4KXR9 were decreased in transgenic plant leaves while being significantly increased after the salt stress treatment.

Also, we found that among the 1082 and 1072 proteins profiled under control and salt stress, 22 (2%) proteins were uniquely found under control conditions, while 11 (1%) were uniquely found after salt stress treatment (Supplementary Figure S8). Among these 11 proteins, G3XDJ4, A0A1Z1CK67, and C8CS23 were adenosine triphosphate (ATP) synthase subunits that are important for the formation of energy storage molecule ATP [32]. Besides, 7 of the 11 proteins including A0A2P5PX19, I1IJ13, A0A1Z1CK67, E9LWD6, I1INX4, and A0A1S3BNT8 were increased after salt stress treatment.

## 3. Discussion 

In this study, we cloned a salt-stress responsive transcription factor *McHB7* from an ice plant with a highly conserved HD and a variable HALZ domain. Using a popular *Agrobacterium*-mediated transformation method [33,34], we were able to obtain transient transgenic ice plants and confirmed OE of the transgene *McHB7* by both qPCR and Western blot after infiltration for 7 and 14 days (Figure 4 and Figure 5). Western blot analysis is important to show increase of *McHB7* without affecting the total protein level [35]. This work was necessary to demonstrate transformation efficiency and genetic manipulation of ice plant. The ability to test gene functions through reverse genetics in calcitrant native system is a breakthrough. Many studies have used heterologous systems—e.g., *A. thaliana* and *Nicotiana benthamiana* (*N. benthamiana*)—for testing gene functions [36,37,38]. While heterologous systems are useful, in vivo research is always more applicable. Interestingly, the transgene expression in the ice plant appears to be long lasting, in contrast to the *N. benthamiana* system that often lasts a short time [39]. Here, OE of the *McHB7* gene in ice plant leaves prolonged for two weeks after infiltration (Figure 5).

Salinity is a major abiotic stress factor that can induce water deficit [40], leading to an accumulation of ROS (e.g., O_2_^−^, H_2_O_2_, and hydroxyl radical (OH^−^)) that damage cell membranes and essential molecules like DNA, proteins, and lipids [41,42]. With the OE ice plants, we examined the different physiological parameters including SOD, POD, MDA, and water content in the WT and OE plants under salt stress treatment. SOD is known to function as a primary O_2_^−^ scavenger [43]. POD is an oxidoreductase that detoxifies H_2_O_2_ [44]. MDA is often used as an indicator of oxidative membrane lipid damage [45]. Water content is used to reflect general stress tolerance and water use efficiency [46]. The results clearly showed that OE of *McHB7* effectively increased the activities of SOD and POD, which reduce ROS levels and damage under salt stress. *McHB7* OE improved plant development and growth under salinity stress by maintaining ion homeostasis and water balance. 

Proteomics analysis suggested that many proteins were increased or decreased in the *McHB7* overexpressing plants compared to WT. For example, K9NCW5 (Rubisco), a key enzyme involved in the first step of carbon fixation in photosynthesis [47]. It was reported that the small subunit of Rubisco was coded by nuclear genes [48,49]. Here we showed that *McHB7* protein was localized within the nucleus and that OE of *McHB7* in ice plant increased the abundance of Rubisco, indicating that *McHB7* is likely a positive regulator of carbon fixation. The abundance of Rubisco in OE plants was significantly elevated under both control and stress conditions, which showed *McHB7* may function in the process of photosynthesis by mediating the expression of the proteins. More experiments are needed to uncover the relationship between *McHB7* and Rubisco. D7KSJ9 is known to play an important role in response to stimulus and regulation [50]. Its protein level is two times higher in OE plants than in the WT before salt stress. After salt stress, its protein level increased about 12 times in the OE plants compared to WT. A0A0D2Q010 is an ATP-citrate synthase beta chain protein and known to participate in the citrate cycle [51]. It was also increased in OE plants after salt treatment. These results suggest that *McHB7* is involved in stress response through regulating stress and energy-related proteins as a master switch. 

Since the ice plant genome has not been published, we used the corresponding Arabidopsis homolog to determine potential HB7 binding sites in the 5-kb upstream promoter of *AtHB7* (AT2G46680.1) by searching PlantCARE (http://bioinformatics.psb.ugent.be/webtools/plantcare/html/) (accessed on 1 May 2021). Several *cis*-acting elements related to light-regulation like the LAMP-element, ACE, GT1-motif, 3-AF1 binding site, Sp1, and chs-CMA1a were found (Supplementary Table S3), which could be bound by numerous proteins [52]. Among these elements GT1-motif was reported in *Rubisco*, which was regulated at the transcriptional level through light-responsive pathway [53]. In addition, numerous stress-responsive elements like an oxidative stress-responsive element *as*-1 were found in the promoter region of *AtHB7*. The binding activity of *as*-1 could be stimulated by oxidative stress [54]. However, salt stress was considered to induce osmotic stress and enhance accumulation of ROS in plant cells [55]. Surprisingly, several proteins that were related to oxidative phosphorylation like AOA078JRB4, B9HTA1, and I1J2V1 were identified in OE plants and the abundances of these proteins were increased compared to WT. This indicates *McHB7* might function as a stress response TF induced by high salinity to enhance the stress tolerance of plants through regulation of oxidative-related proteins. MBS, LTR, and TC-rich repeats were also reported to respond to various abiotic stresses [56]. Some hormone-responsive *cis*-elements including ABRE, GARE-motif, TGACG-motif, P-box, TATC-box, and TCA-element were identified which were known to be responsive to abscisic acid, gibberellin, methyl jasmonate, or salicylic acid [56]. The results suggest that *McHB7* is a signal for stresses dependent on the hormonal signaling pathway. Interestingly, according to our proteomics, we found the protein A0AOJ8BHN7 was identified under control and salt stress conditions and elevated in the OE plants. It is also involved in plant hormone signal transduction based on GO analysis. 

Protein functions may be informed by their interacting networks. The homologous gene of *McHB7* in Arabidopsis is *AtHB7*. They share 41.3% identity at the amino acid level (Supplementary Figure S9A) with high similarity in the HD and HAZL domains. According to String network analysis, four proteins—*At*HB5, RD26, ABI2, and ABF3—were found to be tightly associated with *At*HB7 at high confidence (confidence > 0.7) (Supplementary Figure S9B). Among them, *At*HB5 acts as a positive regulator of ABA-responsiveness and mediates the inhibitory effect of ABA on growth [57]. RD26 encodes a NAC transcription factor and participates in ABA-mediated dehydration response [58]. ABI2 encodes a phosphatase 2C protein and regulates numerous ABA responses [59]. ABF3 encodes an ABA-responsive element-binding protein and can respond to stress and abscisic acid [60]. *At*HB7 is also transcriptionally regulated in an ABA-dependent pathway [61]. It may interact with these ABA-related proteins and mediate the response to adverse environmental effects. In addition, it was reported that ABA-induced *AtHB7* can promote leaf development, chlorophyll synthesis, and reduce stomatal conductance in mature plants [62]. Also, *AtHB12* functions as a positive transcriptional regulator of PP2C genes and plays a vital role in response to water deficit [63]. The proteomics data and String network analysis provides a functional context of *McHB7*, which can be explored further in the future studies.

## 4. Materials and Methods

### 4.1. Plant Materials and Salt Stress Treatment

*Mesembryanthemum crystallinum* seeds were sowed into the moist soil in a growth chamber at 12-h (26 °C) light/12-h dark (18 °C) cycle. Seven days later, the seedlings with four leaves were transplanted to the 946 mL foam cups and watered with 50 mL 0.5× Hoagland’s solution each day. One-month-old ice plants were used for transformation and salt treatment. For high salinity stress, the plants were irrigated with 50 mL 500 mM NaCl in 0.5× Hoagland’s solution every day. The control plants were irrigated with 50 mL 0.5× Hoagland’s solution.

### 4.2. Cloning and Sequence Analysis of McHB7 Gene

A total of 100 mg leaf material from an individual ice plant was collected and ground in liquid nitrogen into a fine powder. Total RNA was extracted according to the instructions of a RNeasy^®^ Plant Mini Kit (QIAGEN, Germantown, MD, USA). The RNA was then reversely transcribed into cDNA following a protocol of ProtoScript^®^ II First Strand cDNA Synthesis Kit (New England BioLabs, Ipswich, MA, USA). Based on the sequence information obtained from RNA-Seq [15], a pair of specific primers *cMcHB7* (Supplementary Table S1) were designed for PCR amplification of the *McHB7* open-reading-frame (ORF). With the NCBI database, the amino acid of *McHB7* was used to blast for homologous proteins. A phylogenetic tree was constructed with MEGA 7 and multi-sequence alignment was analyzed by BioEdit (Version 7.2). Pfam database (http://pfam.xfam.org, accessed on 8 May 2021) was applied for conserved motifs prediction, and online software WebLogo (https://weblogo.berkeley.edu/logo.cgi, accessed on 8 May 2021) was used for motif visualization.

### 4.3. Subcellular Localization of McHB7 Protein

The subcellular localization of *McHB7* protein was predicted by online software CELLO2GO (http://cello.life.nctu.edu.tw/cello2go, accessed on 8 May 2021). Briefly, the amino acid sequence of *McHB7* was pasted to the software in FASTA format, and blast-searched in Eukaryote (E-value 0.001). To confirm the predicted result, the ORF without stop codon of *McHB7* was PCR amplified using a pair of primers *gMcHB7* (Supplementary Table S1), which contains *NcoI* and *Spe I* restriction sites, respectively. Then the PCR fragment was ligated to a plant expression vector pCAMBIA1302 with a green fluorescent protein mGFP5. The recombinant plasmid called *McHB7*-*GFP* was transformed into *Agrobacterium tumefaciens* GV3101. *McHB7*-*GFP* and the positive control pCAMBIA1302 agrobacteria were cultured in a LB broth liquid medium containing 50 mg/mL kanamycin and 25 mg/mL rifampicin to an OD_600_ of 0.8–1.0. The agrobacteria were collected by centrifugation and resuspended in a MES solution containing 100 mM MES (pH 5.8), 100 mM MgCl_2_, and 100 µM acetosyringone (As) and used for transformation. *N. benthamiana* seedlings were grown in the growth chamber conditions, and two-month-old tobacco leaves were used for infiltration with blunt-end micro-syringe [33]. Each leaf was injected with 500 µL *Agrobacterium*, and four replicate experiments were done. Two days later, the GFP signal was observed using a confocal laser scanning microscope (Zeiss, Jena, Germany).

### 4.4. McHB7 Gene Expression Analysis

To investigate the relative expression of *McHB7* in the leaves of ice plant under high salinity conditions, one-month-old ice plant seedlings were irrigated with 50 mL 500 mM NaCl solution each day for 14 days. The second pair of leaves were harvested and midveins discarded for RNA extraction. Each sample has four biological replicates and three technical replicates. Plasma membrane intrinsic protein 1; 2 (*McPIP1*; 2) was used as the internal reference. The 2× SYBR Green qPCR Master Mix kit (Bimake, Houston, TX, USA) was used for RT-qPCR using a CFX96 171 Touch™ Real-Time PCR Detection System (Bio-Rad, Hercules, CA, USA). Real-time primers are listed in Supplemental Table S1. The relative expression in different samples was calculated using the 2^−ΔΔCt^ method [64]. Based on Student’s *t*-test, bars in the figures correspond to standard deviation. A star indicates *p*-value < 0.05, two stars indicate *p*-value < 0.01, and three stars indicate *p*-value < 0.001.

### 4.5. McHB7 Overexpression Vector Contruction and Plant Transient Transformation

Based on the *McHB7* sequence, a pair of primers *pMcHB7* contains *Bam HI* and *Xba I*, respectively, was designed (Supplementary Table S1). The reverse primer contained the sequence of 3× Flag tag. The ORF without stop codon was ligated to an *Agrobacterium* binary vector pCAMBIA1300, which confers kanamycin (Kan) resistance. The recombinant plasmid was transformed into *Agrobacterium tumefaciens* GV3101 for plant transformation and selected on LB medium with 50 mg/mL Kan. A modified *Agrobacterium*-mediated transformation method was used to transform the leaves of four-week-old ice plants [34]. GV3101 with the recombinant plasmid *pMcHB7* was grown in 25 mL LB liquid medium containing 50 mg/mL Kan and 25 mg/mL rifampicin overnight at 28 ℃. The cells were collected by centrifugation at 10,000 rpm for 10 min at room temperature, and then resuspended into 154 mM NaCl solution with 100 µM As. The OD_600_ was adjusted to 0.8–1.0 for infection. The second pair of expanded leaves was injected with 500 µL of the agrobacterial cultures using a needle-less syringe. The control wild type (WT) plants were injected with the same solution without agrobacteria. Seven days later, the injected leaves were harvested for further analyses. Each sample had four replicates.

### 4.6. Protein Extraction and Validation of Transgenic Ice Plant

A phenol method was used for protein extraction [6]. Infiltrated ice plant leaves (1 g fresh weight) were ground into powder by motor and pestle. A total of 3 mL extraction buffer (100 mM Tris-HCl, pH 8.8, 10 mM EDTA, 0.9 M Sucrose, 20 mM 2-Mercaptoethanol, 1× protease inhibitor cocktail, and 1 mM PMSF) and 3 mL Tris-saturated phenol were added into the protein powder, and then transferred to a centrifugation tube, and vortexed at 4 °C for 1 h. The samples were centrifuged at 4 °C, 15,000× *g* for 15 min. The upper layer was transferred to a new tube carefully, then five times volume of 0.1 M ammonium acetate in 100% methanol was added for protein precipitation. The samples were placed in −20 °C overnight. After centrifugation at 4 °C, 15,000× *g* for 15 min, the supernatant was removed. The precipitate was washed, resuspended in 80% acetone, and transferred to a new 2 mL centrifuge tube, placed in −20 °C for 20 min. After centrifugation at 4 °C, 13,000 rpm for 15 min, and washing again with 100% acetone, the protein pellet was dissolved in 150 µL dissolution buffer (6 M Urea, 1 mM EDTA, 1% SDS, and 50 mM Tris-HCl, pH 8.5). Protein quantification was determined by using a Bradford assay (Thermo Scientific, San Jose, CA, USA) following manufacturer’s instructions. Equal amounts of proteins were loaded on a 10% SDS-PAGE gel. Four biological replicates were conducted for each sample.

For Western blot analysis [65,66], proteins in the SDS-PAGE gel were transferred onto a nitrocellulose immobilization membrane (PerkinElmer Life Sciences, Boston, MA, USA) at constant amperage (0.01 A overnight at 4 °C). Protein loading was assessed by Ponceau S staining. The membrane was washed several times by TBST buffer (50 mM Tris base, 150 mM NaCl, 0.1% Tween 20, pH7.4) and blocked by blocking buffer (5% non-fat milk in TBST buffer) for 1 h. Then, the membrane was incubated with a primary anti-Flag monoclonal antibody (Sigma-Aldrich, Saint Louis, MO, USA) that was diluted into 1 mL TBST buffer (at 1:5000 dilution) and 30 mg no-fat milk for 2 h. After washing the membrane five times by TBST, 5 min. each time, the membrane was incubated by a secondary anti-IgG antibody that was diluted into 1 mL TBST (at 1:10,000) for 1 h. The membrane was washed five times in TBST, then incubated with Western blot signal enhancer reagent (Thermo Scientific™ Pierce™ Western Blot Signal Enhancer, Walham, MA, USA) for 5 min and detected by chemiluminescence using an Amersham Imager 600 (GE Healthcare, Marlborough, MA 01752, USA).

### 4.7. Biochemical Analysis of Antioxidative System Components and ROS

Physiological parameters including superoxide SOD, POD, MDA, and water content of transgenic ice plant seedlings and WT control were measured according to previously published methods [67]. Histochemical staining is an important technique for visualizing biological structures and detection of ROS, including O_2_^−^, H_2_O_2_, hydroxyl radical (OH^−^), singlet oxygen, and lipid hydroperoxides [68]. In this study, DAB, NBT, and Evans blue were carried out according to published methods [69,70]. Four biological replicates were conducted for each treatment and a Student’s *t*-test was used for statistical analysis.

### 4.8. Liquid Chromatography Mass Spectrometry (LC-MS/MS) and Data Analysis

The protein samples from four biological replicates of WT control and four biological replicates of transgenic plants were digested with trypsin as previously described [6]. Liquid chromatography tandem mass spectrometry (LC−MS/MS) was carried out on an Easy-nLC 1200 system (Thermo Fisher Scientific Inc., Germering, DE, USA) coupled with a Q-Exactive HF Orbitrap mass spectrometer (Thermo FisherScientific Inc., San Jose, CA, USA). The peptides were separated by an Acclaim PepMap100 C18 column (250 mm × 75 μm; 2 μm-C18) (Thermo Fisher Scientific Inc., San Jose, CA, USA) with 3 h gradient at the flow rate of 0.35 µL/min. The LC gradient was: 0–5 min, 2% B; 140 min, 35% B; 160 min, 100% B; 165 min, 100% B; 170 min, 2% B; 180 min, 2% B, run stop. The MS was operated between MS scan and MS/MS scan automatically with a cycle time of 3 s. Eluted peptides were detected in the Orbitrap MS at a resolution of 120 K with a scan range of 350–1800 m/z, and the most abundant ions bearing 2–7 charges were selected for MS/MS analysis. Automatic gain control (AGC) for the full MS scan was set as 200,000 with maximum injection time (MIT) as 50 ms, and AGC Target of 10,000 and MIT of 35 ms were set for the MS/MS scan. The MS/MS scan used quadrupole isolation mode, high energy collision-induced dissociation (HCD) activation energy, and 35% collision energy, and Orbitrap detection. A dynamic exclusion time of 30 s was applied to prevent repeated sequencing of the most abundant peptides. 

Proteome Discoverer™ 2.4 (Thermo Fisher Scientific, Bremen, Germany) was used for protein identification. The SEQUEST algorithm in the Proteome Discoverer was used to process raw data files. Spectra were searched using the TAIR10 protein database with the following parameters: 10 ppm mass tolerance for MS1 and 0.02 Retention Time tolerance as mass tolerance for MS2, two maximum missed tryptic cleavage sites, a fixed modification of carbamidomethylation (+57.021) on cysteine residues, dynamic modifications of (oxidation of methionine (+15.996) and phosphorylation (+79.966) on tyrosine, serine, and threonine. Search results were filtered at 1% false discovery rate (FDR) and peptide confidence level was set for at least two unique peptides per protein for protein identification. Relative protein abundance in the samples was measured using label-free quantification in the Proteome Discoverer 2.4. Proteins identified and quantified in all 4 out of 4 biological samples were used, and no imputation was performed. Peptides in the control and transgenic samples were quantified as area under the chromatogram peak. The data were normalized by medium and generalized logarithm transformation (Log2). The heatmap was generated using online software MetaboAnalyst 5.0 (https://www.metaboanalyst.ca, accessed on 15 April 2021). Functional categorization was built by Proteome Discoverer software and venn diagram was made by Venny 2.1.0 (https://bioinfogp.cnb.csic.es/tools/venny, accessed on 1 May 2021). Protein network analysis was conducted using String (https://string-db.org/cgi/network, accessed on 1 May 2021).

## 5. Conclusions

In this study, we cloned *McHB7* TF gene from ice plant leaves, and successfully obtained transient *McHB7* OE plants. Under salt stress, ROS and cell death tend to increase in WT. Overexpressing *McHB7* helped to counteract these changes and maintain ROS homeostasis. The results of biochemical and proteomic analyses showed *McHB7* TF exerts a positive effect on the response to high salinity in ice plant. This work provides a quick and effective method for testing gene functions in the native plant species. Identification of genes directly regulated by this salinity responsive TF, as well as its molecular complexes, are interesting future research directions. The findings about *McHB7* may inform molecular breeding and biotechnological efforts towards enhancing crop resilience and productivity.

## Data Availability

The proteomics data have been deposited to the ProteomeXchange Consortium via the PRIDE partner repository with the data set identifier PXD025961 (userID: reviewer_pxd025961@ebi.ac.uk; password: M1rQA2wo).

## Supplementary Materials

The following are available online at https://www.mdpi.com/article/10.3390/ijms22126390/s1. Figure S1: Cloning *McHB7* from ice plant leaves, Figure S2: Subcellular localization prediction with CELLO2GO, Figure S3: Leaf fresh weight measurement of WT and *McHB7* OE plants, Figure S4: Physiological parameters in of WT and *McHB7* OE plants, Figure S5: Leaf fresh weight measurement of WT and *McHB7* OE plants, Figure S6: Relative quantification of levels of H2O2 and O_2_^−^ in ice plant leaves based on NBT and DAB staining, Figure S7: Significantly increased proteins in transgenic ice plant leaves under control and salt stress treatment, Figure S8: Identified proteins under control and salt stress conditions, Table S1: List of primers sequences for cloning and quantitative real-time PCR, Table S2: List of phosphorylated proteins, Table S3: Cis-acting elements in the upstream promoter of AtHB7.
