# Supplementary material for: Proteomics of Homeobox7 Enhanced Salt Tolerance in Mesembryanthemum crystallinum
Source: Int J Mol Sci. 2021 Jun 15;22(12):6390. doi: 10.3390/ijms22126390 (PMC8232686; doi:10.3390/ijms22126390)
Supplement: Supplementary file 1 [file ijms-22-06390-s001.zip › ijms-1241568-supplementary.pdf]

Proteomics of Homeobox7 Enhanced Salt Tolerance in *Mesembryanthemum crystallinum*

Xuemei Zhang [1,2], Bowen Tan [2], Dan Zhu [2,3], Daniel Dufresne [4], Tingbo Jiang [1,*] and Sixue Chen [2,5,6,*]

[1] State Key Laboratory of Tree Genetics and Breeding, Northeast Forestry University, Harbin 150040, China; zhangxuemei199111@gmail.com
[2] Department of Biology, Genetics Institute, University of Florida, Gainesville, FL 32610, USA; tanbowen@ufl.edu (B.T.); zhudan2014dora@163.com (D.Z.)
[3] College of Life Sciences, Qingdao Agricultural University, Qingdao 266109, China
[4] Department of Chemistry, Florida Atlantic University, Boca Raton, FL 33431, USA; dufresne71@yahoo.com
[5] Plant Molecular and Cellular Biology Program, University of Florida, Gainesville, FL 32610, USA
[6] Proteomics and Mass Spectrometry, Interdisciplinary Center for Biotechnology Research, University of Florida, Gainesville, FL 32610, USA
* Correspondence: tbjiang@yahoo.com (T.J.); schen@ufl.edu (S.C.)

Supplementary data

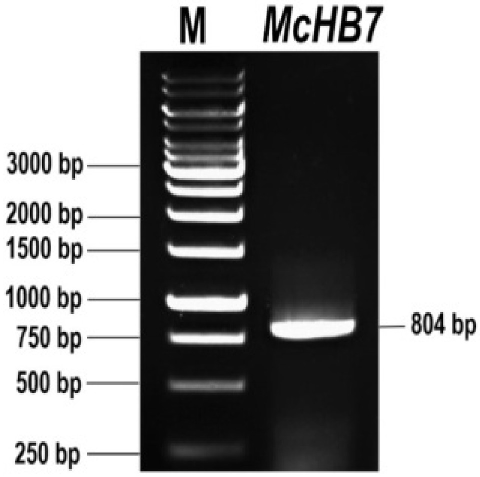

Figure S1 Cloning *McHB7* from ice plant leaves

SeqID: McHB7

Localization Prediction

Localization	Score
Extracellular	0.022
Plasmamembrane	0.026
Cytoplasmic	0.152
Cytoskeletal	0.012
ER	0.016
Golgi	0.010
Lysosomal	0.005
Mitochondrial	0.052
Chloroplast	0.043
Peroxisomal	0.023
Vacuole	0.011
**Nuclear**	**4.626**

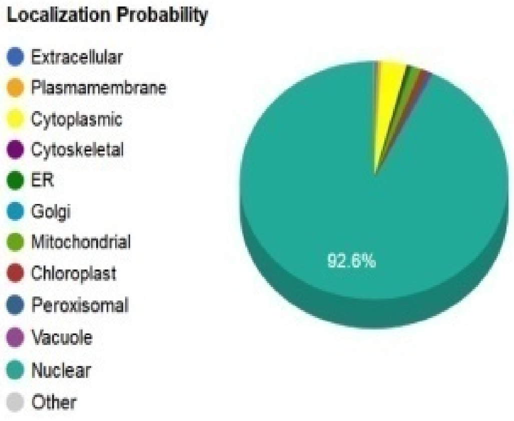

Figure S2 Subcellular localization prediction with CELLO2GO

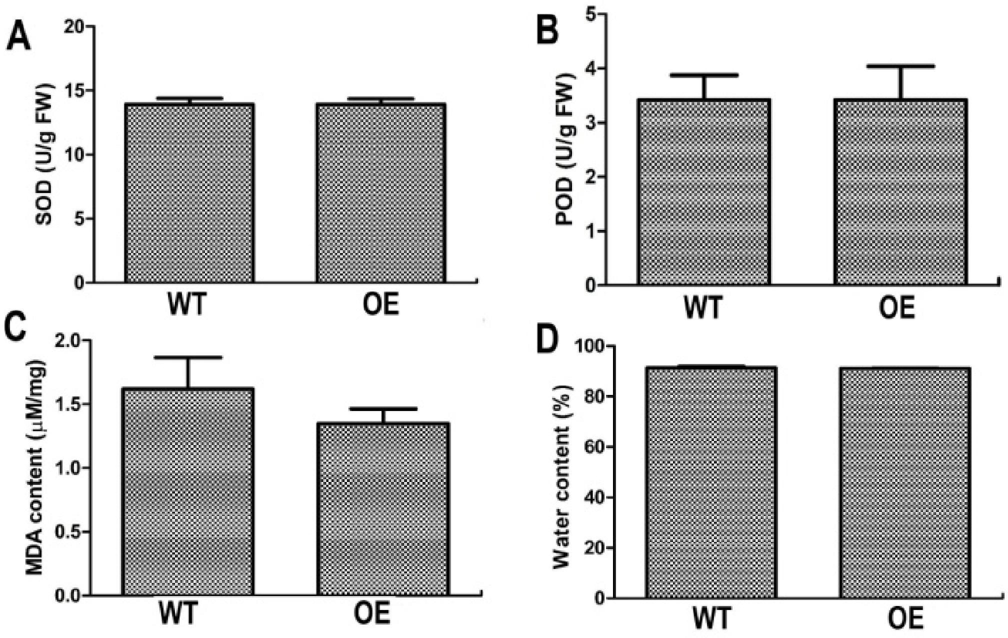

Figure S3 Physiological parameters in OE ice plant. **(A)** SOD activity of OE and WT ice plants. **(B)** POD activity. **(C)** MDA content. **(D)** Water content.

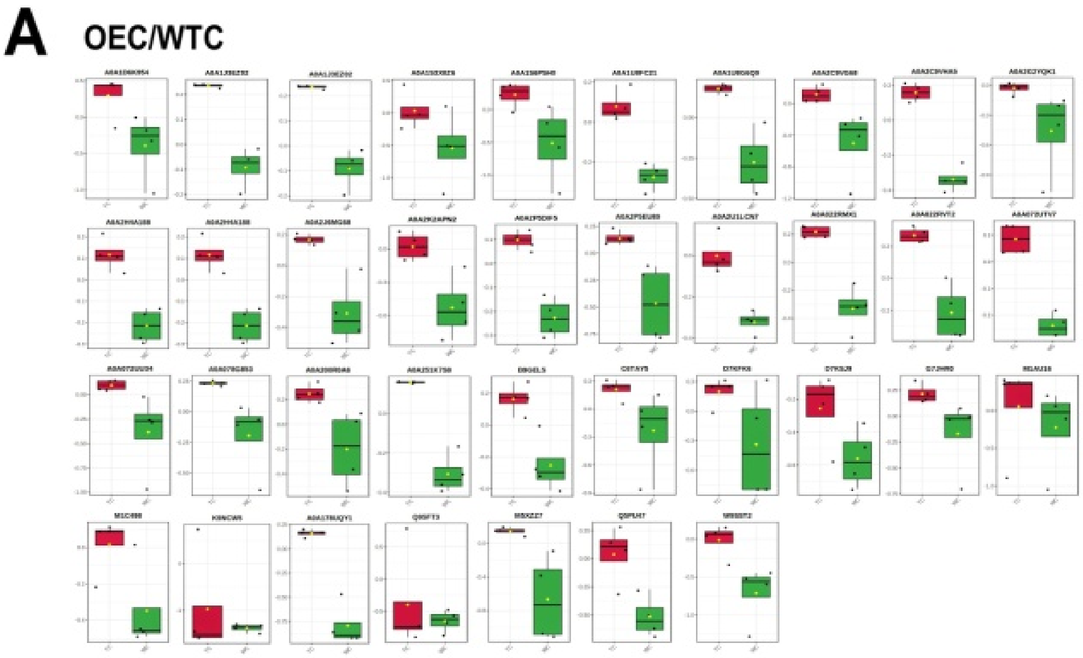

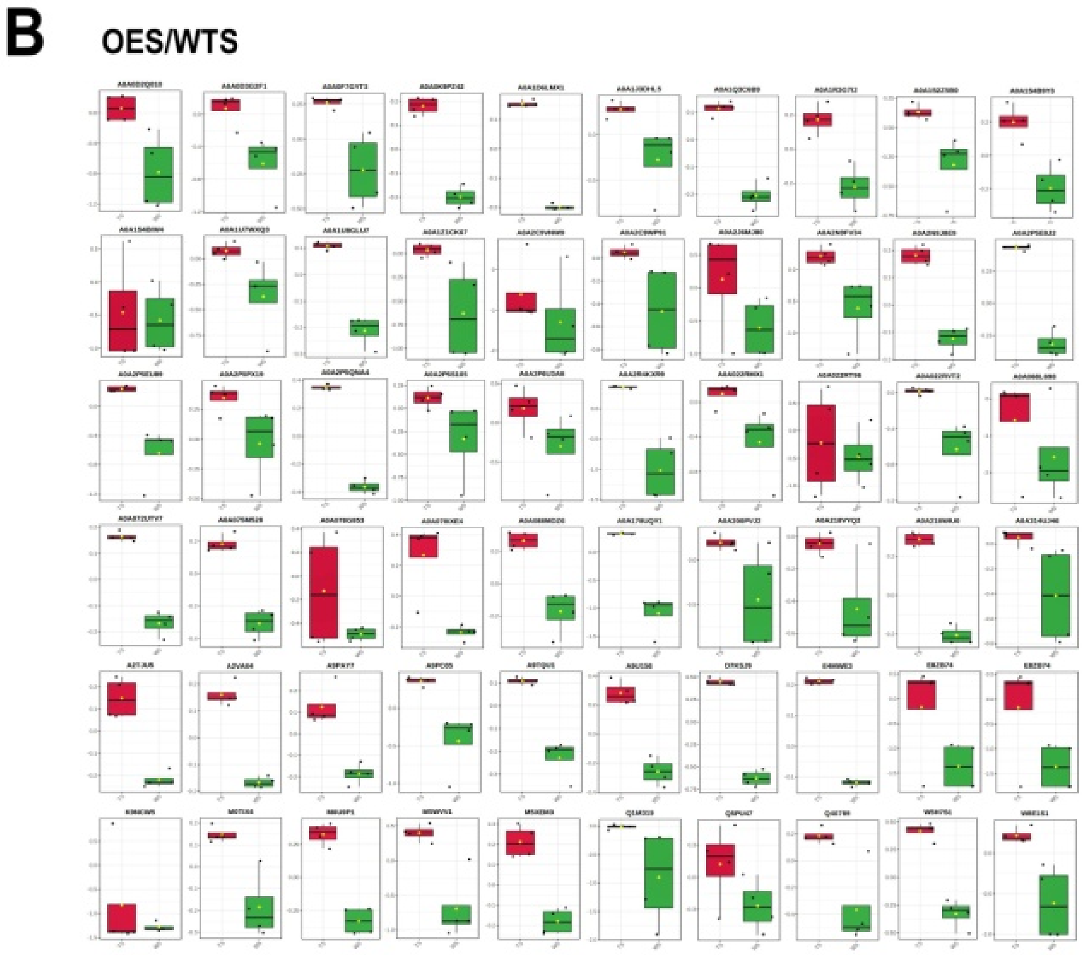

Figure S4 Significantly increased proteins in transgenic ice plant leaves under control and salt stress treatment. **(A)** Increased proteins under control conditions. **(B)** Increased proteins under salt stress conditions

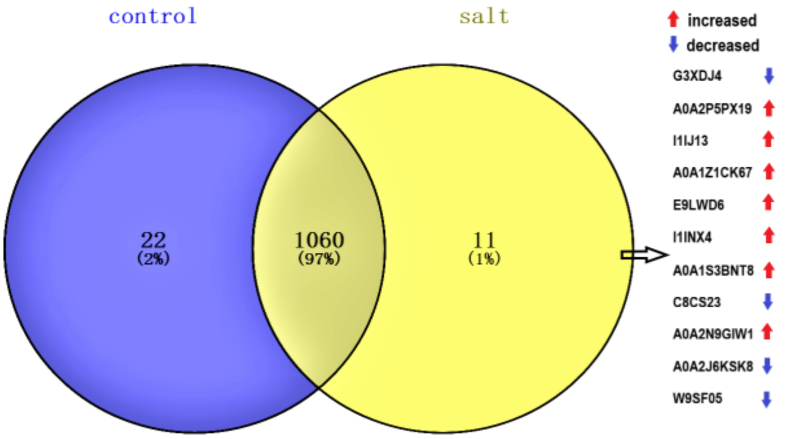

Figure S5 Identified proteins under control and salt stress conditions.

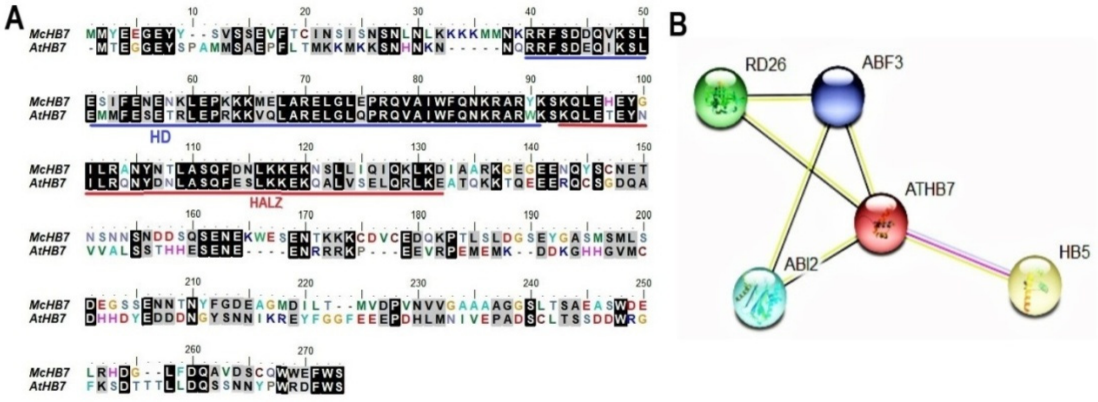

Figure S6 Alignment with *AtHB7* and *McHB7*. **(A)** Alignment of *AtHB7* and *McHB7*. **(B)** Protein-protein interaction network of *AtHB7*.

Table S1. List of primers sequences for cloning and quantitative real-time PCR

Gene name	Primer sequence(5′-3′)
**cMcHB7**	F: ATGATGTATGAAGAAGGAGAA<br>R: TCACGACCAAAATTCCCACCA
**gMcHB7**	F: CGCCCATGGATGATGTATGAAGAAGGAGAA<br>R: CGGACTAGT CGACCAAAATTCCCACCATTG
**McPIP1;2**	F: GATGCCAAGAGGAGTGCTAG<br>R: GTTCCAGTGATTGGGATGGTT
**rtMcHB7**	F: CGAGACGAACAGCAATAATAGT<br>R: CTTCACACACATCGCATTTCT
**pMcHB7**	F: CGCGGATCCATGATGTATGAAGAAGGAGAA<br>R:CGGTCTAGATCAGGCGCCTTTGTCATCGTCATCCTTGTAGTCTCCGCC TTTATCGTCATCGTCTTTATAATCTCCGCCTTTGTCATCGTCATCCTTGTA GTCTCCGCCCGACCAAAATTCCCACCATTG

cMcHB7 was the primer for *McHB7* gene cloning; gMcHB7 was the primer for GFP ligation, the underlines were *NcoI* and *Spe I* restriction sites, respectively; McPIP1;2 was the internal primer for RT-qPCR; rtMcHB7 was the primer for *McHB7*quantification using RT-qPCR; and pMcHB7 was the primer for overexpression vector construction, the underlines were *Bam HI* and *XbaI*, respectively.

Table S2 Posphorylated proteins

Accession	Description	Peptides	Unique Peptides
Q40185	Chlorophyll a-b binding protein	4	2
A0A022RT96	Chlorophyll a-b binding protein	6	2
A0A078II53	Glutamine synthetase	3	2
A0A1D8DAB1	Ribulose bisphosphate carboxylase large chain	2	2
A0A0A0QMA2	Ribulose bisphosphate carboxylase large chain	3	3
B8BPB7	Uncharacterized protein	3	2
A0A1S4CJP2	ribulose bisphosphate carboxylase/oxygenase activase 1, chloroplastic isoform X1	10	2
A0A087H1T9	Glutamine synthetase	2	2
A0A2R6XLV7	Uncharacterized protein	8	2
K4AXU0	Uncharacterized protein	13	2
A0A1Y0KLB7	Ribulose bisphosphate carboxylase large chain	3	2
A0A2P6U3A8	Rubisco activase	3	2
Q2HW53	Ferredoxin-dependent glutamate synthase; Glutamate synthase, large subunit region 1 and 3, putative; Glutamate synthase	14	2
I3SU63	Fructose-bisphosphate aldolase	3	2
B9HKC1	Uncharacterized protein	4	3
A0A2P5AJP3	Chlorophyll a-b binding protein	6	2
A0A1S4BC47	Chlorophyll a-b binding protein	5	3
M7YTH1	Chlorophyll a-b binding protein	8	2
Q41423	Chlorophyll a-b binding protein	5	2
A0A2P5DIF5	Chlorophyll a-b binding protein	6	4
Q9TKH0	Ribulose bisphosphate carboxylase large chain	3	2
A0A0S2LMW5	ATP synthase subunit beta	2	2
W1P4U4	Chlorophyll a-b binding protein	8	2
A0A178VDH1	CSP41A OS=Arabidopsis thaliana	4	2
M5X4I0	Malate dehydrogenase	6	2
K3YTH6	Uncharacterized protein	2	2
G0WYB2	Ribulose bisphosphate carboxylase large chain	2	2
A0A0B5H5B4	Ribulose bisphosphate carboxylase large chain	3	2
A0A1U8G7Q4	phosphoglucomutase	9	3
C5IFT7	Chlorophyll a-b binding protein	5	3
A0A218W3S5	Uncharacterized protein	6	2
B9HRB0	Uncharacterized protein	3	2
A0A2N9J3G3	Chlorophyll a-b binding protein	5	2
M1AAF6	Uncharacterized protein	4	4

Table S3 *Cis*-acting elements in the upstream promoter of *AtHB7*

site name	sequence	function
**MBS**	CAACTG	MYB binding site involved in drought-inducibility
**as-1**	TGACG	an Oxidative Stress-Responsive Element
**WUN-motif**	AAATTTCCT	wound-responsive element
**ABRE**	ACGTG	involved in the abscisic acid responsiveness
**GARE-motif**	TCTGTTG	gibberellin-responsive element
**LAMP-element**	CTTTATCA	part of a light responsive element
**MRE**	AACCTAA	MYB binding site involved in light responsiveness
**GCN4_motif**	TGAGTCA	involved in endosperm expression
**CCAAT-box**	CAACGG	MYBHv1 binding site
**ACE**	CTAACGTATT	involved in light responsiveness
**TGACG-motif**	TGACG	involved in the MeJA-responsiveness
**GATA-motif**	AAGATAAGATT	part of a light responsive element
**GT1-motif**	GGTTAA	light responsive element
**ARE**	AAACCA	*cis*-acting regulatory element essential for the anaerobic induction
**TC-rich repeats**	ATTCTCTAAC	defense and stress responsiveness
**P-box**	CCTTTTG	gibberellin-responsive element
**LTR**	CCGAAA	low-temperature responsiveness
**TATC-box**	TATCCCA	gibberellin-responsiveness
**CAT-box**	GCCACT	related to meristem expression
**3-AF1 binding site**	TAAGAGAGGA A	light responsive element
**TCT-motif**	TCTTAC	part of a light responsive element
**G-box**	TAACACGTAG	involved in light responsiveness
**TCA-element**	CCATCTTTTT	involved in salicylic acid responsiveness
**Box 4**	ATTAAT	part of a conserved DNA module involved in light responsiveness
**O2-site**	GATGATGTGG	involved in zein metabolism regulation
**Sp1**	GGGCGG	light responsive element
**GA-motif**	ATAGATAA	part of a light responsive element
**CGTCA-motif**	CGTCA	involved in the MeJA-responsiveness
**AE-box**	AGAAACAA	part of a module for light response
**chs-CMA1a**	TTACTTAA	part of a light responsive element
**Box II**	ACACGTAGA	part of a light responsive element
